# P056. Clinical case: headache as a symptom of another disease detector

**DOI:** 10.1186/1129-2377-16-S1-A191

**Published:** 2015-09-28

**Authors:** Fortunata Tripodi

**Affiliations:** ASP RC, Reggio Calabria, Italy

GC, 57 years of age, lawyer, smoker (30 cigarettes a day), in treatment for hypertension, referred frequent panic attacks (PAs) in the last two years treated correctly. He had experienced a cerebral ictus about six months before coming to my attention and at that time was hospitalized in the AO Stroke Unit where he had undergone a CT angiography. The symptoms completely regressed after six hours. He came to my attention complaining of a headache for the past three months, oppressive in nature, right frontal-orbital subcontinuous, with no nausea, or vomiting, moderate pain.

Neurological examination: objective examination was normal, and the examinations performed during hospitalization in the Stroke Unit were evaluated.

He emphasized that he felt very stressed and was afraid of what had happened to the point that he has noticed an increase in panic attacks. A psychological evaluation was performed confirming the anxiety syndrome thus, an anxiolytic was added to his medication therapy of SSRIs. He was asked to fill in a headache diary and return for a follow-up visit after 30 days. At follow-up, blood tests were normal and the situation was unchanged. Considering the presence of a headache that did not seem to have the characteristics of any particular form and was unclassifiable, PAs were re-evaluated. The patient referred a sudden difficulty in breathing and required a deep breath during the ictal episode. He lost consciousness and after recovering from this state reported paresis of the left side lasting about six hours.

It was hypothesized that perhaps the incidents described were not PAs but could be critical partial episodes, not stroke, with a generalization of a crisis with post-critical paralysis. Therefore, an MRI and EEG were repeated and resulted abnormal (figure 1).

**Figure 1:**
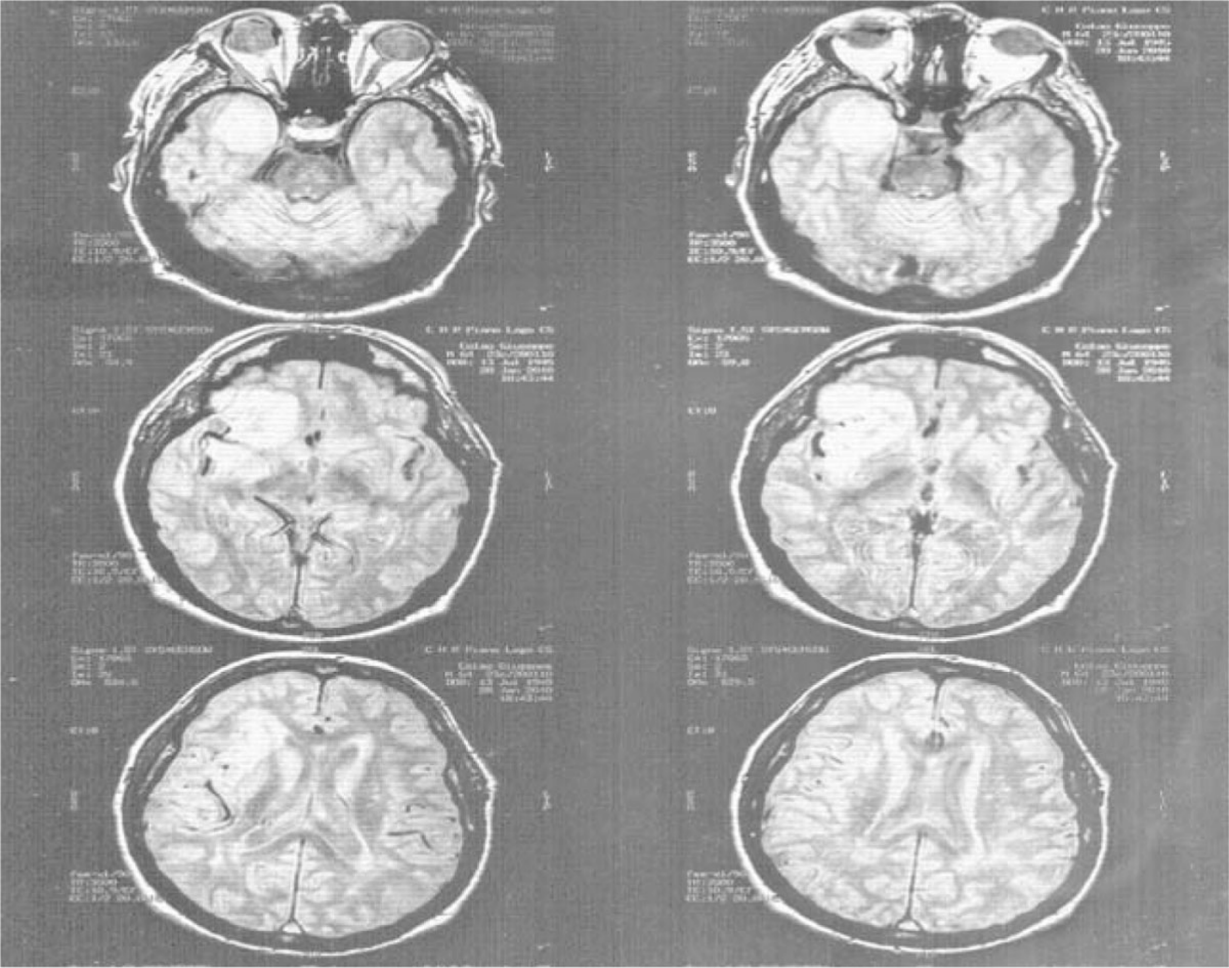

He was diagnosed with symptomatic headache and the histological diagnosis revealed oligodendroglioma.

## Discussion

This tumor could be found in all the locations of the hemispheres of the brain, although the frontal and temporal are the most common locations. Oligodendrogliomas are generally soft textured and have a gray-pink structure. They contain minerals deposits (calcifications) with areas of hemorrhage and/or cysts. Under a microscope, cancer cells appear with “short arms or a “fried egg” shape.

Written informed consent to publish was obtained from the patient(s).

